# Predictive blood plasma biomarkers for EGFR inhibitor-induced skin rash

**DOI:** 10.18632/oncotarget.17060

**Published:** 2017-04-12

**Authors:** Vivien Hichert, Catharina Scholl, Michael Steffens, Tanusree Paul, Christian Schumann, Stefan Rüdiger, Stefan Boeck, Volker Heinemann, Volker Kächele, Thomas Seufferlein, Julia Stingl

**Affiliations:** ^1^ Research Division, Federal Institute for Drugs and Medical Devices, Bonn, Germany; ^2^ Institute of Pharmacology of Natural Products and Clinical Pharmacology, University of Ulm, Ulm, Germany; ^3^ Department of Internal Medicine II, University of Ulm, Ulm, Germany; ^4^ Pneumology, Thoracic Oncology, Sleep and Respiratory Critical Care Medicine, Clinics Kempten-Oberallgäu, Kempten, Germany; ^5^ Department of Internal Medicine III and Comprehensive Cancer Center, Ludwig-Maximilians-University of Munich, Munich, Germany; ^6^ Department of Internal Medicine I, University of Ulm, Ulm, Germany; ^7^ Centre for Translational Medicine, University Bonn Medical Faculty, Bonn, Germany; ^8^ DKTK, German Cancer Consortium, German Cancer Research Center, (DKFZ), Heidelberg, Germany

**Keywords:** EGFR inhibitor-induced skin rash, predictive biomarkers, amphiregulin, hepatocyte growth factor, calcidiol

## Abstract

Epidermal growth factor receptor overexpression in human cancer can be effectively targeted by drugs acting as specific inhibitors of the receptor, like erlotinib, gefitinib, cetuximab and panitumumab. A common adverse effect is a typical papulopustular acneiform rash, whose occurrence and severity are positively correlated with overall survival in several cancer types. We studied molecules involved in epidermal growth factor receptor signaling which are quantifiable in plasma, with the aim of identifying biomarkers for the severity of rash. With a predictive value for the rash these biomarkers may also have a prognostic value for survival and disease outcome.

The concentrations of amphiregulin, hepatocyte growth factor (HGF) and calcidiol were determined by specific enzyme-linked immunosorbent assays in plasma samples from 211 patients.

We observed a significant inverse correlation between the plasma concentration of HGF and overall survival in patients with an inhibitor-induced rash (p-value = 0.0075; mean overall survival low HGF: 299 days, high HGF: 240 days) but not in patients without rash. The concentration of HGF was also significantly inversely correlated with severity of rash (p-value = 0.00124).

High levels of HGF lead to increased signaling via its receptor MET, which can activate numerous pathways which are normally also activated by epidermal growth factor receptor. Increased HGF/MET signaling might compensate the inhibitory effect of epidermal growth factor receptor inhibitors in skin as well as tumor cells, leading to less severe skin rash and decreased efficacy of the anti-tumor therapy, rendering the plasma concentration of HGF a candidate for predictive biomarkers.

## INTRODUCTION

The epidermal growth factor receptor (EGFR, also HER1) belongs to the ErbB family of receptor tyrosine kinases. It regulates fundamental cell functions, like survival, proliferation and migration, via numerous signaling pathways including the mitogen-activated protein kinase (MAPK) cascade (Ras/RAF/MEK/ERK), the phospholipase C (PLCγ/PKC) and the Akt (PI3K/Akt) pathways [1]. EGFR is often over-expressed or over-activated in human cancer [2, 3]. Frequently administered inhibitors which are specific for EGFR (EGFRIs) are the tyrosine kinase inhibitors (TKIs) erlotinib and gefitinib and the monoclonal antibodies (mAbs) cetuximab and panitumumab. Additional approved inhibitors include necitumumab (targets EGFR), pertuzumab (targets HER2, prevents dimerization with EGFR), lapatinib (targets EGFR, HER2) and afatinib (targets EGFR, HER2, HER4) [4–7]. For metastatic colorectal cancer, mutations in exons 2/3/4 of the GTPases *KRAS* and *NRAS* are negative predictive biomarkers for efficacy of cetuximab and panitumumab [8]. In non-small cell lung cancer (NSCLC) driver mutations of *EGFR* are positive predictive biomarkers for efficacy of erlotinib and gefitinib [9]. A common adverse effect induced by all EGFRIs is skin toxicity, including xerosis, hair and nail abnormalities and most often a typical papulopustular acneiform rash [10, 11]. Occurrence and severity of the EGFRI-induced skin rash have been shown in several independent studies to be positively correlated with patients’ outcome [12, 13] and have been tested as surrogate marker for drug efficacy and suitable dosing [14–16]. The rash usually reaches its maximal manifestation two to three weeks after initiation of therapy [10]. Common recommendations for management of the rash are topical corticosteroids, topical and oral antibiotics and antihistamines (reviewed in [17]). Hence, its severity may be suppressed, rendering it unsuitable as a clinical predictive marker. Rapidly determinable predictive biomarkers for the severity of EGFRI-induced rash would allow to start early with preventive treatment of the rash and still allow prediction of EGFRI efficacy. Such biomarkers might indicate whether clinicians should intensify therapy and monitoring (e.g. by more frequent tumor imaging). We previously showed that the concentration of interleukin-8 (IL-8) [18] and a metabolic ratio for erlotinib (erlotinib concentration divided by O-desmethyl-erlotinib concentration) [19] could be valuable indicators for the severity of rash and were associated with patients’ survival. A predictive biomarker allows for an in-advance evaluation of the efficacy of a therapy. A prognostic biomarker allows for an in-advance evaluation of the outcome of a disease independent of therapy [20]. To find more reliable biomarkers, we used a candidate approach and selected proteins which are involved in EGFR signaling, can be rapidly measured in patient plasma and have shown first promising results in previous (screening) studies as potential biomarkers for the development of EGFRI-induced rash (amphiregulin and HGF). We also included a completely new promising target (calcidiol).

EGFR is stimulated by various ligands. Amphiregulin is particularly interesting with regard to EGFRI-induced rash because it is known to mediate skin homeostasis by activating keratinocyte proliferation [21, 22]. It is the most abundant EGFR ligand present in cultured human keratinocytes with over seven times more soluble protein than any of the other ligands [23]. Neutralization of amphiregulin with specific antibodies results in significant inhibition of keratinocyte proliferation and decreased phosphorylation of the MAPK extracellular signal-regulated kinase (ERK). Ishikawa et al. previously observed a significant correlation between high serum concentrations of amphiregulin and poor response to gefitinib in patients with NSCLC [24].

Hepatocyte growth factor (HGF) might also influence EGFR signaling via cross-talk of signaling pathways. It is the direct ligand of the receptor tyrosine kinase MET (also called c-MET) and has been found to induce resistance to EGFR inhibitors [25]. HGF is also called scatter factor and it is a cytokine expressed by mesenchymal cells. Activation of MET can lead to an activation of the same pathways which are also activated via EGFR (MAPK, PLCγ and PI3K/Akt pathways) [26]. A synergistic effect of MET and EGFR activation on cell proliferation and motility of NSCLC cells has been found. Also a synergistic effect of MET and EGFR inhibition on apoptosis was shown [27]. This suggests a cross-talk between the two pathways. Hammond and colleagues found a high degree of overlap of effector molecules which were phosphorylated (indicating activation) by epidermal growth factor (EGF) as well as HGF [28]. In 2015 Takahashi and colleagues found a correlation between serum levels of HGF and occurrence of EGFRI-induced skin toxicity in metastatic colorectal cancer (inverse correlation) [29]. We now investigated whether this correlation could also be seen in a larger cohort of patients suffering from either lung, pancreatic, head and neck or colorectal cancer.

Vitamin D_3_ (cholecalciferol/calciol), or rather its main active metabolite calcitriol, can bind to the nuclear vitamin D receptor (VDR), which forms heterodimers with the retinoic X receptor (RXR) [30]. Subsequently, the whole complex specifically binds to vitamin D responsive elements (VDREs), which can be found in the promoters of various target genes. Their transcription is then increased or decreased through complex interactions with numerous co-factors. A putative VDRE has also been found in the *EGFR* promoter region (GGGTCCAGAGGGGCA), which was shown to bind VDR in an RXR-dependent manner in electrophoretic mobility shift assays (EMSAs) [31–33]. Another VDRE has been found in intron 1 of the *EGFR* gene (AGTTGAATAAGTTGA) and its functionality was confirmed in gene reporter analyses in ovarian cancer cells [34]. An increasing effect of calcitriol on EGFR mRNA and protein levels has been observed in osteoblast-like cells [31] and a decreasing effect in some breast cancer cells [33]. If calcitriol can regulate the expression of EGFR it might also increase/decrease the effect of EGFRIs. Interestingly, the enzyme calcidiol-1α-hydroxylase is not just expressed in the kidney but also at several extrarenal tissues, including skin (basal keratinocytes and hair follicles) [35]. It has been shown *in vitro* as well as *in vivo* that human keratinocytes can produce substantial amounts of active calcitriol [36, 37]. The concentration of the storage form of vitamin D, calcidiol, is easily determined in plasma by commercially available enzyme-linked immunosorbent assays (ELISAs) [38].

Our study aimed at identifying biomarkers in patient plasma, which are predictive for the development of EGFRI-induced skin rash and therefore also for response to EGFRI therapy. Plasma biomarkers which can easily and rapidly be measured would save valuable time in determining the efficacy and safety of an EGFRI therapy for an individual patient and also allow preventive treatment of the rash.

## RESULTS

### Skin rash and survival

**Figure 1:**
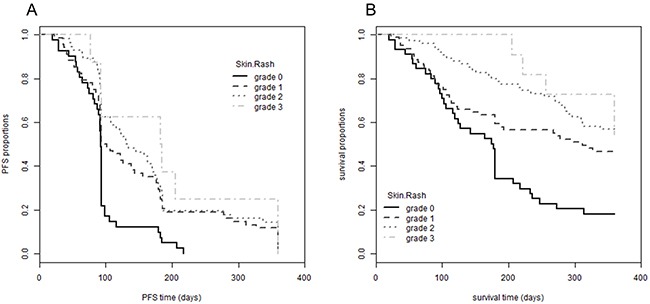
Association between EGFRI-induced skin rash and progression-free and overall survival Patients were followed-up for 360 days after initiation of EGFRI therapy. Patients are grouped according to the maximal grade of skin rash developed during observation period (grade 0 to 3). **(A)** PFS proportion is plotted over the observation period; log rank test, p-value = 2.20 × 10^-4^; mean PFS for grade 0 95 days (SE: 6.7), grade 1 148 days (SE: 12.4), grade 2 165 days (SE: 13.1), grade 3 195 days (SE: 37.4). **(B)** OS proportion is plotted over the observation period; log rank test, p-value = 1.05 × 10^-5^; mean OS for grade 0 180 days (SE: 16.1), grade 1 241 days (SE: 14.6), grade 2 289 days (SE: 11.6), grade 3 324 days (SE: 18.1). PFS and OS times are restricted with an upper limit = 360 days. Abbreviations: EGFRI, epidermal growth factor receptor inhibitor; OS, overall survival; PFS, progression-free survival, SE, standard error.

A cohort of 211 cancer patients was prospectively included in this study according to a protocol described earlier [39]. There were 77 (36.5 %) female and 134 (63.5 %) male participants with a median age of 69 years (range: 43-87 years). Tumor types differed among the patients, with 122 (57.8 %) suffering from non-small cell lung, 46 (21.8 %) from pancreatic, 35 (16.6 %) from colon and 8 (3.8 %) from head and neck cancer. During an EGFRI treatment period of four weeks, 45 patients (21.3 %) developed no skin toxicity while 80 patients (37.9 %) experienced grade 1, 75 (35.5 %) grade 2 and 11 (5.2 %) even grade 3 skin rash (Table 1). No significant association between the severity of skin rash and gender, age or tumor type was observed (data not shown). However, as also reported previously [18, 19], we found a significant correlation between the severity of skin rash and progression-free survival (PFS, p-value = 2.20 × 10^-4^) as well as overall survival (OS, p-value = 1.05 × 10^-5^) of the patients across all four different EGFRIs in our study population (Figure 1).

**Table 1:** Summary of patient characteristics

Patient characteristics	Category	Count (Total: n = 211)	%
Sex	Female	77	36.5
	Male	134	63.5
Age median [years] (range)		69 (43-87)	
BMI median [kg/m^2^] (range)		25.0 (14.2-48.8)	
Smoking status	never	75	35.5
	former	103	48.8
	present	26	12.3
	unknown	7	3.3
Tumor	lung ca	122	57.8
	colon ca (*KRAS* WT)	35	16.6
	head and neck ca	8	3.8
	pancreatic ca	46	21.8
EGFRI applied	erlotinib^1^	133	63.0
	(100 mg)	(45)	
	(150 mg)	(84)	
	gefitinib	11	5.2
	cetuximab	61	28.9
	panitumumab	6	2.8
Maximal skin rash during observation period (grades after NCI-CTCAE)	0	45	21.3
	1	80	37.9
	2	75	35.5
	3	11	5.2

^1^ Indicated standard erlotinib dose depends on tumor type.

Abbreviations: BMI, body mass index; ca, cancer; EGFRI, epidermal growth factor receptor inhibitor; NCI-CTCAE, National Cancer Institute - Common Terminology Criteria for Adverse Events; WT, wild type.

### Association between plasma concentrations of growth factors and overall survival

**Figure 2:**
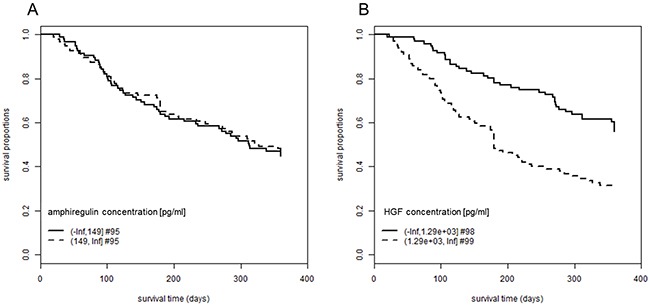
Association between plasma concentrations of amphiregulin and HGF and overall survival Patients were followed-up for 360 days after initiation of EGFRI therapy. The proportion of patients still alive is plotted over the observation period. Patients are grouped according to their plasma concentration of **(A)** amphiregulin, log rank test, p-value = 0.78, mean OS for low amphiregulin concentrations: 252 days (SE: 12.5), mean OS for high amphiregulin concentrations: 255 days (SE: 12.7) and **(B)** HGF, log rank test, p-value = 1.4 × 10^-5^, mean OS for low HGF concentrations: 290 days (SE: 10.6), mean OS for high HGF concentrations: 210 days (SE: 12.4). OS times are restricted with an upper limit = 360 days. Abbreviations: #, number of patients; EGFRI, epidermal growth factor receptor inhibitor; HGF, hepatocyte growth factor; OS, overall survival; SE, standard error.

The plasma concentrations of the EGFR ligand amphiregulin and the MET ligand HGF at four weeks after initiation of EGFRI treatment were determined and tested for association with OS of the patients. For amphiregulin plasma levels between 5 and 1303 pg/ml (median: 150 pg/ml) were measured and for HGF plasma levels between 167 and 17580 pg/ml (median: 1292 pg/ml). The concentration of amphiregulin was not significantly correlated with OS (p-value = 0.78) while the correlation of the concentration of HGF with OS was significant (p-value = 1.4 × 10^-5^). A mean survival time of 290 days (standard error (SE): 10.6) was calculated for patients with low HGF levels (≤ 1290 pg/ml) and 210 days (SE: 12.4) for patients with high levels (> 1290 pg/ml) (Figure 2). We also conducted subgroup analyses for the different tumor entities and the correlation between HGF levels and OS was significant for the two larger groups NSCLC (n = 122; p-value = 0.0012) and pancreatic cancer (n = 41; p-value = 0.00014). Only for the other two groups head and neck cancer (n = 6; p-value = 0.59) and colon cancer (n = 28; p-value = 0.63) the subgroup sizes were too small to detect the effect (data not shown).

**Figure 3:**
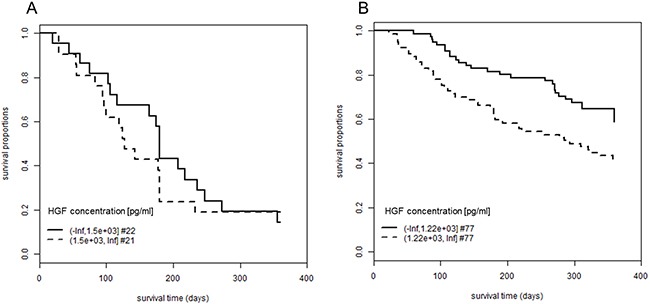
Association between plasma concentration of HGF and overall survival in patients with or without skin rash Patients were followed-up for 360 days after initiation of EGFRI therapy. The proportion of patients still alive is plotted over the observation period. Patients are grouped according to their plasma concentration of HGF. **(A)** Patients who did not develop EGFRI-induced skin rash; log rank test, p-value = 0.56; mean OS for low HGF concentrations: 193 days (SE: 22.5), mean OS for high HGF concentrations: 164 days (SE: 23.6). **(B)** Patients who developed EGFRI-induced skin rash (grades 1 to 3); log rank test, p-value = 0.0075; mean OS for low HGF concentrations: 299 days (SE: 11.2), mean OS for high HGF concentrations: 240 days (SE: 14.5). OS times are restricted with an upper limit = 360 days. Abbreviations: #, number of patients; EGFRI, epidermal growth factor receptor inhibitor; HGF, hepatocyte growth factor; OS, overall survival; SE, standard error.

We subdivided the patient cohort into two groups, according to whether they had developed an EGFRI-induced skin rash or not. This allowed to investigate whether the increased OS in patients with low plasma concentrations of HGF was specific for patients with EGFRI-induced skin rash or rather a general observation for all patients (Figure 3). The association was only significant in the subgroup of patients with skin rash (p-value = 0.0075) but not for the ones without rash (p-value = 0.56). In the subgroup of patients with skin rash, the mean OS time was 299 days (SE: 11.2) for patients with low HGF levels (≤ 1220 pg/ml) and 240 days (SE: 14.5) for patients with high levels (> 1220 pg/ml).

### Correlation between plasma concentrations of amphiregulin and HGF and EGFRI-induced skin rash

**Figure 4:**
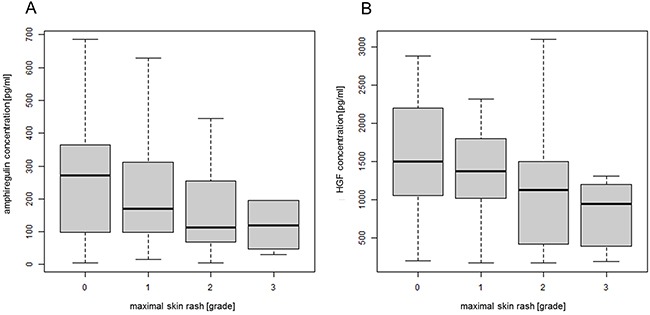
Correlation between plasma concentrations of amphiregulin and HGF and EGFRI-induced skin rash The plasma concentrations of amphiregulin and HGF were determined by ELISA (amphiregulin n = 190; HGF n = 197). **A**) Amphiregulin concentration plotted against the maximal grade of skin rash developed during observation period; Linear Trend Test, p-value = 0.0763. Outliers (> 700 ng/ml) were not included in the figure but still used in the calculations. **(B)** HGF concentration plotted against the maximal grade of skin rash developed during observation period; Linear Trend Test, p-value = 0.00124. Outliers (> 2750 ng/ml) were not included in the figure but still used in the calculations. Abbreviations: ELISA, enzyme-linked immunosorbent assay; EGFRI, epidermal growth factor receptor inhibitor; HGF, hepatocyte growth factor.

Because the association between HGF level and OS of the patients was only significant in patients who had developed EGFRI-induced skin rash, we further analyzed the correlation of the plasma concentrations of HGF and amphiregulin with this rash. There seemed to be a trend for increased severity of rash in patients with low plasma concentrations of amphiregulin (Figure 4A). However, the correlation did not reach significance (p-value = 0.0763). The plasma concentration of HGF was significantly inversely correlated with skin rash (p-value = 0.00124) (Figure 4B).

### Correlation between plasma concentration of calcidiol and EGFRI-induced skin rash

The plasma concentration of calcidiol was also measured at four weeks after initiation of EGFRI treatment and tested for association with OS. Calcidiol is the storage form of vitamin D and measurement of its plasma concentration is most suitable to detect vitamin D status. In our study the plasma levels varied between 0.3 and 84.0 ng/ml (median: 19.7 ng/ml; mean: 21.3 ng/ml). In the literature there is no standardized cut-off value defining vitamin D sufficiency and deficiency. However, most studies suggest that plasma levels of ≥ 20 ng/ml are sufficient for calcidiol to have a beneficial effect on health, e.g. with regard to bone mineral density and cancer-related as well as all-cause mortality [40, 41]. Therefore, in our study we defined calcidiol levels ≤ 20 ng/ml as low and > 20 ng/ml as high levels. When comparing OS times between patients with low and those with high calcidiol levels, there was no significant difference.

**Figure 5:**
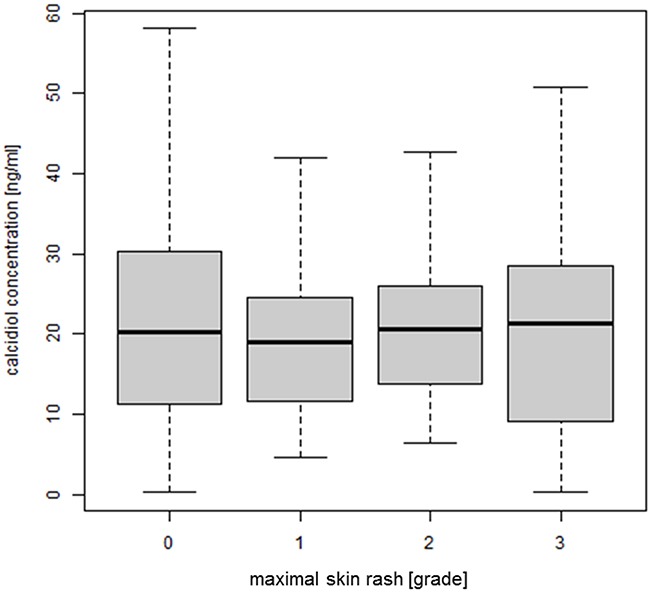
Correlation between plasma concentration of calcidiol and EGFRI-induced skin rash The concentration of calcidiol was determined by ELISA (n = 211) and plotted against the maximal grade of skin rash developed during observation period; ANOVA, p-value = 0.415. Abbreviations: ANOVA, analysis of variance; ELISA, enzyme-linked immunosorbent assay; EGFRI, epidermal growth factor receptor inhibitor.

In addition, the plasma concentration of calcidiol did not significantly correlate with the severity of rash (p-value = 0.415) (Figure 5).

## DISCUSSION

Occurrence and severity of EGFRI-induced skin rash show a positive correlation with patient outcome, rendering this rash a potential marker for drug efficacy [12, 13, 16]. The rash is often treated with systemic or topical medication, which decreases its severity and therefore also its suitability as clinical marker for treatment efficacy or survival. In this study we assessed several substances in patients’ plasma for their suitability as predictive biomarkers for EGFRI-induced skin rash and treatment efficacy. We found that in our patient population the plasma concentration of the MET ligand HGF was inversely correlated with OS of the patients (p-value = 1.4 × 10^-5^). This result confirms the observations reported by Takahashi and colleagues in 2014. They analyzed serum levels of different growth factors in patients with metastatic colorectal cancer who were treated with an anti-EGFR antibody and also found a significant inverse correlation between serum levels of HGF and OS [42]. Our results show that the correlation between HGF levels and OS is not restricted to patients suffering from colorectal cancer and being treated with mAbs. It can also be observed in a patient cohort including different types of cancers (lung, pancreatic, head and neck and colon cancer) and EGFRIs (mAbs and TKIs).

It is well established that over-activated HGF/MET signaling increases invasive growth and metastasis by inducing motility of tumor cells and survival in remote tissue sites [43, 44]. This effect could explain why higher levels of HGF are associated with decreased survival and it would rather render HGF a prognostic biomarker. However, the correlation between plasma concentration of HGF and OS was only significant in the subgroup of patients who had developed skin rash and not in the group without rash. This suggests that HGF might rather have a predictive implication for the efficacy of EGFR inhibition. We also observed a significant inverse correlation between HGF levels and severity of EGFRI-induced rash. This finding is in accordance with the Takahashi study from 2015, which also found that patients with higher serum levels of HGF developed lower grades of skin toxicity [29]. They measured a median HGF concentration of 1337 pg/ml in serum which is in accordance with our median of 1292 pg/ml determined in plasma. The mechanism behind the correlation between HGF levels and skin rash is not completely understood so far. HGF/MET signaling has been linked to resistance to EGFRI therapy in many types of cancer [25], like NSCLC (gefitinib) [45, 46] and colon cancer (cetuximab) [47]. MET can activate a number of pathways which are normally also activated by EGFR, like the MAPK, PLCγ and PI3K/Akt pathways [26]. It is probable that in the complex signaling networks containing EGFR and MET, certain effectors or even pathways are redundant for certain functions under specific physiological conditions [28, 43]. When EGFR is inhibited in cancer therapy, tumor cells possibly evade death by increasing MET signaling. This compensation of EGFR inhibition by HGF/MET signaling might also happen in skin cells and could explain why higher plasma concentrations of HGF correlate with less severe EGFRI-induced skin rash. In 2007 Spix and colleagues found that in human corneal epithelial cells HGF activates downstream effectors of EGFR, like ERK1/2, via MET and it could also stimulate EGFR itself via activation of EGFR ligands, like amphiregulin [48]. The group also found indications for these mechanisms to be present in human epidermal keratinocytes.

HGF is a candidate predictive biomarker for the efficacy of EGFRI therapy. It should be noted that in our subgroup of patients who developed no skin rash a trend towards an association between plasma levels of HGF and OS was also visible. Even though this association was not significant, it might indicate that the plasma concentration of HGF is not exclusively predictive for therapy efficacy but also prognostic for patients’ outcome (OS), independently of the activity of the EGFRI. It has been shown in a previous study in metastatic colorectal cancer patients that the occurrence of EGFRI-induced skin rash was significantly associated with OS in patients with mutations in codon 12 of *KRAS* in tumor cells [49]. Such a mutation leads to an EGFR-independent activation of the MAPK pathway, rendering EGFRIs ineffective and making it surprising that EGFRI-induced rash was still associated with OS. This suggests that, in addition to being predictive for EGFRI efficacy, the skin rash might also partially be a prognostic marker, which would match our observations for HGF.

In our patient samples the plasma concentration of the EGFR ligand amphiregulin was not significantly correlated with OS or the occurrence of EGFRI-induced skin rash. This finding is in contrast to the results by Takahashi and colleagues, who found that patients with higher serum levels of amphiregulin developed lower grades of skin toxicity [29]. This discrepancy in results could be due to the fact that they analyzed pre-treatment samples while in our study plasma samples were only available from four weeks after initiation of the EGFRI therapy. In previous studies the group around Takahashi showed that the serum levels of amphiregulin increased during EGFRI therapy in 95 % of patients. HGF levels only increased in 58 % of patients and also to a much lesser extent [42]. This might also explain why we measured higher concentrations of amphiregulin in our cohort (5-1303 pg/ml) than Takahashi (3-636 pg/ml) and Ishikawa (10-380 pg/ml), who both used pre-treatment serum samples for their analyses [24, 29]. Our results show that HGF is more stable as a biomarker than amphiregulin over the course of therapy. However, it might be advisable to use plasma taken prior to the start of the EGFRI therapy.

To our knowledge we were the first group to investigate the correlation of calcidiol levels on the development of EGFRI-induced rash. In our cohort we measured a mean calcidiol plasma concentration of 21 ng/ml, which is slightly lower than the mean concentration measured by He and colleagues (33 ng/ml) [38] which can be due to differences in the used assay kits. There was no association between the plasma concentration of calcidiol and the severity of EGFRI-induced skin rash. VDREs have been identified in the promoter region [31–33] and intron 1 [34] of the *EGFR* gene and calcitriol was shown to either increase or decrease EGFR mRNA and protein levels in different cell types [31, 32]. However, a possible activation/inhibition of EGFR in skin cells by calcitriol does not seem to be strong enough to measure an according increase/decrease in severity of EGFRI-induced rash.

Our study population has the limitation that all patients received an EGFRI and there was no control group with a different therapy. This renders it difficult to draw definite conclusions about purely prognostic or predictive biomarkers. However, if the concentration of a certain substance is correlated with outcome in patients who developed skin rash but not in the ones with no rash, it can be assumed that this parameter is rather predictive for EGFRI efficacy. It also has to be noted that we focused our study on biomarkers for efficacy of inhibition of EGFR signaling, irrespective of the type of EGFR inhibitor used. Different types of EGFR inhibitors might have different additional off-target effects and respective additional predictive/prognostic biomarkers, which cannot be identified in our patient cohort.

Taken together, we confirmed HGF as a tentative predictive biomarker, which in our cohort was predictive for the efficacy of EGFRI therapy irrespective of the tumor site. Its plasma concentration showed significant inverse correlations with the severity of EGFRI-induced skin rash and OS in patients who developed the rash. Future studies including a control group with patients not treated with an EGFRI will help to further clarify the role of HGF as a predictive or prognostic biomarker, also confirming in larger cohorts if the predictive value is independent of the specific tumor site. In addition, the collection of pre-treatment plasma samples might allow for more conclusive results about the potential of amphiregulin plasma concentration as biomarker. Our results for HGF indicate that the HGF/MET pathway is interesting with regard to its role for EGFRI efficacy and we suggest other proteins from this pathway as promising new targets for future research. Additional targets, like for example miRNAs, are also conceivable. The identification of reliable biomarkers predictive for the efficacy of EGFRI therapy and the establishment of rapid and reproducible assays with high sensitivity to measure the plasma concentrations would be of high value for the improvement of EGFRI cancer therapy for individual patients.

## MATERIALS AND METHODS

### Patient samples

Plasma samples (n = 211) were derived from patients included in the Dermatoxgen study, which is a prospective, multicenter study designed to investigate pharmacogenetic factors of skin toxicity induced by EGFR inhibitors, as already reported in previous publications [18, 19, 39]. The study includes patients with histologically confirmed solid tumors (pancreatic, colon, head and neck or non-small cell lung cancer) who are first-time treated with an EGFRI (erlotinib, gefitinib, cetuximab or panitumumab). All included colon cancer patients have wild type *KRAS*. Written informed consent was obtained from all patients and the study was approved by the ethical boards of Ulm University and the Ludwig-Maximilians-University of Munich. An EGFRI was administered according to approved indication, either alone or in combination with various chemotherapeutic agents. Erlotinib was applied daily at a dose of 100 mg (n = 46) in pancreatic cancer or 150 mg (n = 84) in NSCLC (for one patient dose unknown). For two additional patients the dose was reduced to 50 mg during the course of erlotinib therapy (n = 2). Gefitinib was also applied daily but at a dose of 150 mg (n = 1) or 250 mg (n = 10). A dose of 250 mg/m^2^ of cetuximab was administered weekly (n = 58) or bi-weekly (n = 3). A dose of 6 mg/kg body weight of panitumumab was administered bi-weekly (n = 6).

The development of skin rash and other adverse effects was monitored once a week over a treatment period of four weeks. The severity of skin rash was graded according to the Common Toxicity Criteria for Adverse Events of the American National Cancer Institute (NCI CTCAE version 3.0, 2006) [50]. Patients received reactive treatment for rash as necessary during course of therapy, including topical corticosteroids, topical antibiotics, antihistamines and oral antibiotics.

At the fifth visit (four weeks after initiation of EGFRI therapy) a blood sample was drawn from each patient and plasma samples were generated at the respective study site. Follow-up visits were conducted at three, six and twelve months after start date of treatment. The survival status at 360 days after initiation of EGFRI treatment was used for Kaplan-Meier analyses.

### Preparation of plasma samples

Blood samples were collected four weeks after initiation of EGFRI therapy right before application of the next scheduled dose. Five patients treated with erlotinib were on a therapy break prior to sample collection (2 patients for 5 days, 1 patient for 6 days, 2 patients for 8 days). From each patient 7.5 ml blood were collected in a blood sampling tube containing anti-coagulant (S-Monovette^®^ EDTA, 7.5 ml, Sarstedt). The samples were centrifuged at 1992 x g and 4 °C for 10 min. The supernatants (plasma) were collected and immediately transferred to -20 °C in aliquots. For long-term storage the samples were kept at -80 °C.

### Enzyme-linked immunosorbent assays (ELISAs)

The plasma concentrations of amphiregulin were determined using the Human Amphiregulin ELISA Kit from Sigma-Aldrich (cat. no. RAB0019-1KT), which includes an anti-amphiregulin capture antibody, a biotinylated anti-amphiregulin detection antibody, a horseradish peroxidase linked to streptavidin as enzyme and 3,3’,5,5’-tetramethylbenzidine (TMB) as substrate. The concentrations of HGF were determined using the Quantikine® ELISA Human HGF from R&D systems (cat. no.: DHG00), which includes a monoclonal anti-HGF capture antibody, a polyclonal anti-HGF antibody linked to the enzyme horseradish peroxidase and TMB as substrate. The concentrations of calcidiol were determined using the 25-OH-Vitamin-D-ELISA from EUROIMMUN Medizinische Labordiagnostika AG (cat. no.: EQ 6411-9601), which includes a monoclonal antibody specific for cholecalcidiol and ergocalcidiol as capture antibody, peroxidase-linked streptavidin for detection and TMB as substrate. All ELISAs were conducted according to the respective manual of the manufacturer. All samples were measured in duplicates and 4-parameter curve fits were used for analyses.

Plasma concentration of amphiregulin was successfully measured for 190, HGF for 197 and calcidiol for 211 patients. For some of the 211 patients the volume of available plasma sample was not sufficient to adequately perform all three types of ELISAs.

### Statistical analysis

Plasma concentrations of the different analytes (amphiregulin, HGF, calcidiol) were tested for association with the multinomial end-point maximal skin rash using analysis of variance (ANOVA) and the Jonckheere-Terpstra linear trend test. In case the endpoints were dichotomized associations were calculated by Student's t-test.

Survival distributions between different patient groups were compared using Kaplan-Meier analysis. OS times were restricted with an upper limit = 360 days. Data from patients lost to follow-up were censored at the day last known alive. Due to the nature of our censored data, the non-parametric log-rank test was used. All p-values are reported as nominal p-values and p-values < 0.05 were regarded as significant. Since some of the survivor functions do not fall below a survival portion of 0.5, median OS and 95 % confidence intervals cannot be calculated. Therefore, mean OS times with standard error are provided for comparisons.

Statistical analyses were performed with R v3.2.5 including the libraries coin v1.1-2 and survival 2.39-5 (R Foundation for Statistical Computing, Vienna, Austria).

We also thank Kerstin Brandenburg for assistance with the conduction of HGF and calcidiol ELISAs and Bärbel Reiser for management of the clinical data.
